# Associations between Child Mental Well-Being or Conflicts during Mealtime and Picky Eating Behaviour

**DOI:** 10.3390/ijerph18115621

**Published:** 2021-05-25

**Authors:** Maria Lepinioti, Ina Olmer Specht, Jeanett Friis Rohde, Maria Stougaard, Mina Nicole Händel, Nanna Julie Olsen, Berit Lilienthal Heitmann

**Affiliations:** 1 The Parker Institute, Research Unit for Dietary Studies, Bispebjerg and Frederiksberg Hospital, DK 2000 Frederiksberg, Denmark; ina.olmer.specht@regionh.dk (I.O.S.); jeanett.friis.rohde@regionh.dk (J.F.R.); maria.stougaard@psy.ku.dk (M.S.); mina.nicole.holmgaard.handel@regionh.dk (M.N.H.); nanna.julie.olsen@regionh.dk (N.J.O.); Berit.lilienthal.heitmann@regionh.dk (B.L.H.); 2 The Boden Institute of Obesity, Nutrition, Exercise & Eating Disorders, The University of Sydney, Sydney, NSW 2050, Australia; 3 Department of Public Health, Section for General Medicine, University of Copenhagen, DK 1014 Copenhagen, Denmark

**Keywords:** pickiness, child behavior, mealtime, conflicts, preschool children

## Abstract

Pickiness is an eating behavior that many families with young children face. Having joint family meals may impact the child’s pickiness, for instance by influencing their willingness to try novel foods. Moreover, picky children have been shown to display greater emotionality. The aim of this study was to investigate if children’s mental well-being and parent-reported conflicts during mealtime were associated with pickiness among obesity-prone children. Data was obtained from the baseline examination of the Healthy Start intervention study, the Danish Medical Birth registry and the Danish Health Visitor’s Child Health Database, and included 635 children aged 2–6 years that were all at high risk for becoming overweight later in life. Children’s mental well-being was measured by the strengths and difficulties questionnaire. Crude and adjusted ordinal logistic regressions were used to investigate the cross-sectional associations. Children had a higher odds associated with changing from a category of less pickiness to a category of more pickiness for each one point higher SDQ score (ORadj. = 1.35, 95% CI = 1.14; 1.61) and lower odds (ORadj. = 0.57, 95% CI = 0.38; 0.86) associated with changing pickiness category towards more pickiness for each one point higher SDQ prosocial score. Moreover, children with conflicts during mealtime had higher odds of being in a worse pickiness category compared to children without conflicts (ORadj. = 3.37, 95% CI = 2.27; 5.01). This study showed that among obesity-prone children, behavioral problems, as well as conflicts during mealtime, were associated with more picky behaviors. Further longitudinal studies are needed to confirm the findings, as are studies including general child population subsets.

## 1. Introduction

Picky eating is generally defined as the rejection of a substantial number of foods that are familiar or unfamiliar, but a clear definition is missing [1]. Pickiness is an eating behavior that many families with young children face [2,3]. Picky children show greater emotionality, as well as negative reactions, to food [4,5]. In addition, picky eating has been associated with a range of adverse behavioral outcomes, both externalizing and internalizing [6]. However, there is still uncertainty about the potential causes and health consequences of being picky during childhood [7,8].

Pickiness has been broadly characterized by traits such as inadequate dietary diversity [1,9,10], rejection of a specific food texture [1,11,12] and/or category [10], strong food preferences [2], restricted food intake [2,10] and, according to some studies, also food neophobia [1]. Frequent joint family meals and the home environment, for example conflicts during meals, have been found to have an impact on children’s eating behaviors and can influence children’s willingness to try novel foods or foods they disliked in the past [13,14]. In this regard, making time for joint family meals has been found to be associated with family closeness and to contribute to the emotional well-being of all family members [15,16].

In addition to the frequency of joint family meals, a variety of parental feeding practices associated with pickiness have recently been studied [17,18,19,20,21,22]. Some studies have indicated that pickiness is associated with disrupted meals, however, there are cases where pickiness seemed to be the source, not the consequence, of meal disruption [23]. Furthermore, family mealtimes can be disrupted by parental stress induced by a variety of factors, e.g., related to work or to household issues [24]. When picky children are pressured to eat particular foods, there may also be a greater risk of conflicts at mealtimes, which may be further associated with adverse child behaviors [25]. Contrarily, positive interactions between the parent and child may promote smooth-flowing routines and a healthier diet [26]. 

In the present study, we aimed to investigate if child behavioral problems, measured by the strengths and difficulties questionnaire (SDQ) and the prosocial behavior scale, or conflicts during mealtimes were associated with childhood pickiness.

## 2. Materials and Methods

The present study was based on baseline data collected from the Healthy Start Intervention Study, which was a randomized controlled primary prevention intervention conducted between 2009 and 2011 in 11 municipalities around the greater Copenhagen area. The Healthy Start intervention study aimed to improve diet and physical activity habits, reduce stress and improve sleep quality and quantity over a period of 15 months to prevent excessive weight gain among 2–6-year-old obesity-prone children. The Healthy Start intervention study, including the methodology and eligibility criteria, has been described in detail elsewhere [23]. 

### 2.1. Study Population

The Healthy Start intervention study used the Danish Medical Birth Register [24] and administrative birth forms to identify those children from the 11 municipalities that were born in 2004–2007 and were considered obesity-prone based on them having either a high birth weight (>4000 g) and/or a mother who was overweight prior to pregnancy (body mass index (BMI) > 28 kg/m^2^) and/or a mother with a low educational level (≤10 years). Initially 3058 children were found eligible and were invited to participate in the intervention. The study population included a total of 635 children (543 with healthy weight and 92 who were overweight) who accepted the invitation and agreed to participate. From the baseline data collection of the Healthy Start intervention study, we had information on children’s picky-eating behaviors, SDQ or conflicts during mealtime on 583 children. In the analysis of SDQ and pickiness, five children had missing information, and in the analysis of conflicts during mealtime and pickiness, 16 children had missing information and were thus excluded. Furthermore, due to missing data on the covariates, 17 individuals were excluded from the analysis of SDQ and pickiness, and 35 individuals were excluded from the analyses on conflicts during mealtime and pickiness.

### 2.2. Measures

Parents were asked to complete a questionnaire with general information about the child and family environment at baseline. 

#### 2.2.1. Exposures

##### Strengths and Difficulties Questionnaire

The strengths and difficulties questionnaire (SDQ) is a brief instrument developed to screen for child and adolescent behavior; it consists of 25 items on prosocial behavior and emotional and behavior problems. The single-sided version of the SDQ for 2–4-year-olds was used for the Healthy Start intervention study. The SDQ asks about 25 attributes, some positive and others negative. These 25 items are divided into 5 scales (“Emotional symptoms”, “Conduct problems”, “Hyperactivity/inattention”, “Peer relationship problems” and “Prosocial behavior”). The scores within all categories except “Prosocial behavior” are summed to a total difficulties score (SDQ-TD score), based on a scoring syntax available from the SDQ; results range from 0 to 40, with a higher score corresponding to a worse SDQ (25). The score on the prosocial behavior scale (PSB score) is not incorporated into the SDQ-TD score, as absence of prosocial behaviors differs conceptually from the presence of psychological difficulties [26]. The PSB score was hence used as an exposure variable by itself, with a range of 0 to 10, with a higher scores corresponding to fewer prosocial difficulties. 

##### Conflicts during Mealtime

Information on conflicts during mealtime was based on the question: “How would you describe the meals with the child?” with answers: “very conflicting”, “a bit conflicting”, “very cozy”, “a little cozy” and “do not know”. In the analysis, the answers were dichotomized as conflicting (“very conflicting”, “a bit conflicting”) or not conflicting (“very cozy”, “a little cozy”). 

##### Covariates

Factors thought to be related to child mental well-being or conflicts during mealtime and pickiness were obtained from the parental questionnaire completed by the parents at baseline and from The Danish Medical Birth Registry. Covariates included children’s age (grouped into 2–3 and 4–6 years), sex (boy/girl), as well as their appetite assessed by parents (good appetite, normal, too small). This data was collected by health visitors and originated from the Danish Health Visitors’ Child Health Database. The Database Steering Committee provided the data. Maternal education was obtained from the completed questionnaire and formed three categories: ‘No academic training’ (low), ‘Academic training for up 3–4 years’ (medium) and ‘University degree’ (high). 

### 2.3. Outcome

Information on “pickiness” was based on the question “how would you describe your child’s way of eating?” The parents could answer if they perceived their child as being “picky”, “a little picky” or “likes everything”.

### 2.4. Statistical Analyses

To investigate the differences between pickiness and the covariates, chi-squared tests and a one-way ANOVA were used. Crude and adjusted associations between the SDQ difficulties and prosocial scores, as well as conflicts during mealtimes and picky-eating behaviors, were investigated using ordinal logistic regression. Crude analyses were performed, then adjusted for the potential confounders described above (age, gender). The SDQ-TD and PSB total scores were analyzed as a continuous exposure variable and adjusted for gender and age. The association between conflicts during mealtime (yes/no) and pickiness was additionally adjusted for the SDQ-TD score. Results in the SDQ, PSB and conflicts categories were stratified by the age groups 2–3 years and 4–6 years for further investigation. In a sensitivity analysis, we excluded the children (*n* = 9) with psychiatric diagnoses. 

A two-sided *p*-value of *p* ≤ 0.05 was considered the level of significance. All statistical analyses were carried out using the statistical software SAS/STAT (release 9.4; SAS Institute, Cary, NC, USA).

## 3. Results

Table 1 shows child characteristics according to picky-eating behavior. No differences were observed between picky, a little picky and nonpicky children in relation to sex. It was observed that children between the ages 2–3-years-old liked almost everything (46.8%) whereas those among the ages 4–6-years-old were described as picky (19.1%) (*p* < 0.007). Almost half of the children who were described as picky eaters by their parents were also described as children with a small appetite (*p* < 0.001), whereas the majority (65.1%) who were considered to like almost everything were also considered to have a good appetite (*p* < 0.001) (Table 1). 

The authors observed that more than 75% of the families that reported conflicts during mealtimes also referred to their child as either picky (31.6%) or a little picky (44.4%). In families with no conflicts during mealtimes, only 8.5% of the children were described as picky (*p* < 0.0001). From the results of the SDQ-TD score, it was observed that a higher mean SDQ-TD score was associated with children being pickier (*p* < 0.002). From the SDQ PSB score, the researchers found that a higher mean score was associated with less pickiness among the children (*p* < 0.04) (Table 1).

As shown in Table 2, the odds associated with changing from a category of less pickiness to a category of more pickiness were higher for each one point higher SDQ-TD score (ORadj. = 1.35, 95% CI = 1.14; 1.61). Odds were lower (ORadj. = 0.57, 95% CI = 0.38; 0.86) for changing pickiness category towards more picky when the SDQ PSB score was increased by one point (Table 2). When removing children with psychiatric diagnosis (*n* = 9), odds for ranging from not picky to picky in relation to the SDQ-TD were crude OR: 1.32, 95% CI = 1.11; 1.57 and adjusted OR: 1.34, 95% CI = 1.12; 1.59 (results not in table).

Finally, children with conflicts during mealtime had higher odds (ORadj. = 3.37, 95% CI = 2.27; 5.01) of being in a worse pickiness category compared to children without conflicts (Table 3). Results in both categories were similar when stratified by the age groups, 2–3 years and 4–6 years. When removing children with psychiatric diagnoses, the odds yielded crude: 3.86, 95% CI = 2.61; 5.70 and OR adj: 3.51, 95% CI = 2.35; 5.23.

## 4. Discussion

The present study examined whether child behavior problems and conflicts during mealtime were related to pickiness and found that children with a higher SDQ score were pickier, whereas children who were more prosocial were less picky. Moreover, we found that conflicts during mealtime were also related to picky eating. 

Currently, minimizing picky eating is centered around parental feeding practices [2,15,27,28] and on diminishing parental anxiety [29,30]. Studies have shown that parental modeling and food availability influence children’s eating behavior [13,14,31]. The results of this study present new information supporting that children with conflicts during mealtime are pickier than children with no conflicts. In a study by Cole and colleagues, of a population of 497 preschool-aged children, a higher sense of “positive climate” during family meals was associated with lower odds of picky-eating behavior one year later [32]. Furthermore, Jansen et al. found that having structured family mealtimes was associated with more food enjoyment, as well as less fussy and emotional eating among 462 children aged between 21–27 months [33].

Our findings show that children who are prosocial, suggesting they are helpful and considerate of the feelings of others, are also less picky. Our results indicated that children with more behavioral problems were pickier. Similar results was found in a Portuguese study of 959 children aged from 1.5- to 6-years-old, wherein children that were picky scored higher on all emotional and behavioral problems on the child behavior checklist, both internalizing and externalizing, compared to nonpicky children [34]. Additionally, a study on 1272 Swedish preschool-aged picky eaters in all weight status groups showed higher scores of emotional undereating [35]. Similarly, another study including 913 preschool children found that shyness, emotionality and lower levels of sociability predicted increases in pickiness over the study period [36]. Finally, a study by Pliner et al., including 162 children aged 5 to 11, found that reluctance to try unfamiliar foods was associated with shyness and emotionality [8].

### Strengths and Limitations

A strength to this study is that we used a validated behavioral screening questionnaire—the SDQ—to screen children’s mental health [26]. 

There are also a number of limitations to this study. It can be argued that the use of parent-reported picky eating might be different from reporting by registered professionals and limit psychometric validity. The use of a simple unvalidated question to measure pickiness and conflicts during mealtime rather than a multi-item validated questionnaire could also be considered a limitation and could lead to misclassification that might have influenced our results because of the parent’s perception of their child’s behavior. 

Also, associations between conflicts during mealtime and pickiness can be bidirectional (conflicts leading to pickiness and pickiness leading to conflicts), which because of the cross-sectional study design cannot be examined further in the present study. All participating children were of Danish origin and from a society with generally high affluence. Moreover, the study population was selected based on specific inclusion criteria based on potential neonatal risk factors for being overweight or obese later in life, hence our findings may not generalize to children from less affluent populations, other cultures or children that are not at high risk of later obesity.

## 5. Conclusions

Overall, our results suggest that, among Danish preschool children aged 2–6 years, those with more behavioral problems also seem to be pickier. Moreover, even if children in the present study were generally within the normal SDQ range, a higher prosocial score was found to be associated with less pickiness. Finally, children with conflicts during mealtimes were also found to be pickier compared to children without conflicts. Future research is needed to confirm our findings, for instance in less affluent families of children who are not obesity-prone, and to investigate if other aspects of family environment may be related to children’s picky eating. Thus, due to the selected study population, the results of the present study should be generalized with caution only, and further longitudinal studies are needed to confirm the present findings.

## Figures and Tables

**Table 1:** Child characteristics according to picky-eating behavior.

	*n*	Picky (%)	*n*	A Little Picky (%)	*n*	Likes Almost Everything (%)	*p*-Value
Sex							0.09
Girl	43	17.0	96	37.9	114	45.1	
Boy	43	13.0	154	46.7	133	40.3	
Age in years							0.007
2–3	31	10.5	126	42.7	138	46.8	
4–6	55	19.1	124	43.1	109	37.9	
Appetite							<0.001
Good appetite	6	3.5	54	31.4	112	65.1	
Normal	48	14.1	170	49.9	123	36.1	
Too small	28	48.3	22	37.9	8	13.9	
SDQ difficulties score, mean (SD)	85	8.0 (4.2)	249	6.7 (3.8)	244	6.3 (3.8)	<0.002
SDQ prosocial score, mean (SD)	86	7.3 (2.1)	249	7.7 (1.8)	244	7.8 (1.7)	<0.04
Conflicts during mealtime							<0.0001
Yes	42	31.6	59	44.4	32	24.1	
No	37	8.5	186	42.9	211	48.6	
Maternal education							0.93
High	53	24.4	125	51.7	58	24.0	
Medium	61	24.5	124	49.8	64	25.7	
Low	18	20.9	47	54.7	21	24.4	

Note: Given as column % unless otherwise indicated. *p* value according to χ2 test. Note: The total score range is from 0–40 in the difficulties scores (high score = bad) and 0–10 in the prosocial scores (high score = good).

**Table 2:** Odds for ranging from not picky to picky in relation to the SDQ-TD and the PSB.

	*n*	OR Crude	95% CI	*n*	OR Adj ^2^	95% CI
SDQ total difficulties score ^1,3^	578	1.06	1.02; 1.11	573	1.35	1.14; 1.61
SDQ total difficulties score ^1,3^ (ages 2–3 years) *	291	1.06	1.00; 1.12	291	1.06	1.00; 1.14
SDQ total difficulties score ^1,3^ (ages 4–6 years) *	287	1.07	1.02; 1.14	287	1.07	1.02; 1.14
SDQ prosocial score ^1^	579	0.89	0.82; 0.97	577	0.57	0.38; 0.86
SDQ prosocial score ^1^ (ages 2–3 years) *	293	0.88	0.78; 0.99	293	0.88	0.78; 0.99
SDQ prosocial score ^1^ (ages 4–6 years) *	286	0.88	0.77; 0.99	286	0.88	0.77; 1.00

^1^ Continuous exposure variable. ^2^ Adjusted for sex and age. ^3^ Difficulties scores ranging from 0–27 (less to more). * Stratified analyses based on 2–3 and 4–6-year-old children. Note: Results are given for the entire study population and divided into age groups *.

**Table 3:** Odds associated with changing from one picky category to the next in relation to conflicts during mealtime.

	OR Crude	95% CI	*n*	OR Adj ^1^	95% CI
Conflicts during mealtime					
No (*n* = 434)	1	-		1	-
Yes (*n* = 133)	3.72	2.53; 5.47	562	3.37	2.27; 5.01
No (ages 2–3 years, *n* = 62) *	1	-		1	-
Yes (ages 2–3 years, *n* = 226) *	2.85	1.64; 4.95	262	3.46	2.34; 5.14
No (ages 4–6 years, *n* = 71) *	1	-		1	-
Yes (ages 4–6 years, *n* = 208) *	4.48	2.61; 7.67	278	4.05	2.34; 7.03

^1^ Adjusted for sex, age and SDQ difficulties score. * Stratified analyses based on 2–3 and 4–6-year-old children. Note: Results are given for the entire study population and divided into age groups *.

## Data Availability

Data on the Helathy Start could be found: In order to protect sensitive patient information, all data has been deposited in The Danish National Archives and is available upon online request through http://dda.dk/catalogue/22248, accessed on 17 May 2021. Archive number: 22248.
